# The effect of coronavirus infection (SARS-CoV-2, MERS-CoV, and SARS-CoV) during pregnancy and the possibility of vertical maternal–fetal transmission: a systematic review and meta-analysis

**DOI:** 10.1186/s40001-020-00439-w

**Published:** 2020-09-04

**Authors:** Kuma Diriba, Ephrem Awulachew, Eyob Getu

**Affiliations:** Department of Medical Laboratory Sciences, Health Science and Medical College, Dilla University, Dilla, Ethiopia

**Keywords:** Coronavirus, Novel coronavirus-2019, Infection, Pregnancy, Vertical transmission, Middle East respiratory syndrome, Severe acute respiratory syndrome, Severe acute respiratory syndrome coronavirus-2

## Abstract

**Background:**

Coronavirus is challenging the global health care system from time to time. The pregnant state, with alterations in hormone levels and decreased lung volumes due to a gravid uterus and slightly immunocompromised state may predispose patients to a more rapidly deteriorating clinical course and can get a greater risk of harm for both the mother and fetus. Therefore, this systematic review was aimed to assess the effect of coronavirus infection (SARS-CoV-2, MERS-CoV, and SARS-CoV) during pregnancy and its possibility of vertical maternal–fetal transmission.

**Methods:**

A systematic search was conducted on PubMed, Web of Science, Embase, Google Scholar and the Cochrane Library until the end of April. All authors independently extracted all necessary data using excel spreadsheet form. Only published articles with fully accessible data on pregnant women infected with SARS-CoV, MARS-CoV, and SARS-CoV-2 were included. Data on clinical manifestations, maternal and perinatal outcomes were extracted and analyzed.

**Result:**

Out of 879 articles reviewed, 39 studies involving 1316 pregnant women were included. The most common clinical features were fever, cough, and myalgia with prevalence ranging from 30 to 97%, while lymphocytopenia and C-reactive protein were the most common abnormal laboratory findings (55–100%). Pneumonia was the most diagnosed clinical symptom of COVID-19 and non-COVID-19 infection with prevalence ranged from 71 to 89%. Bilateral pneumonia (57.9%) and ground-glass opacity (65.8%) were the most common CT imaging reported. The most common treatment options used were hydroxychloroquine (79.7%), ribavirin (65.2%), and oxygen therapy (78.8%). Regarding maternal outcome, the rate of preterm birth < 37 weeks of gestation was 14.3%, preeclampsia (5.9%), miscarriage (14.5%, preterm premature rupture of membranes (9.2%) and fetal growth restriction (2.8%). From the total coronavirus infected pregnant women, 56.9% delivered by cesarean, 31.3% admitted to ICU, while 2.7% were died. Among the perinatal outcomes, fetal distress rated (26.5%), neonatal asphyxia rated (1.4%). Only, 1.2% of neonates had apgar score < 7 at 5 min. Neonate admitted to ICU was rated 11.3%, while the rate of perinatal death was 2.2%. In the current review, none of the studies reported transmission of CoV from the mother to the fetus in utero during the study period.

**Conclusion:**

Coronavirus infection is more likely to affect pregnant women. Respiratory infectious diseases have demonstrated an increased risk of adverse maternal obstetrical complications than the general population due to physiological changes occurred during pregnancy. None of the studies reported transmission of CoV from the mother to the fetus in utero, which may be due to a very low expression of angiotensin-converting enzyme-2 in early maternal–fetal interface cells.

## Background

Coronaviruses (CoVs) are one of the major pathogens that are grouped in the family of Coronaviridae, which primarily target the human respiratory system [1]. It is one of the emerging and reemerging viral outbreaks throughout the world. Previous outbreaks of coronaviruses include the severe acute respiratory syndrome (SARS)-CoV epidemic in 2003 [2] and the Middle East respiratory syndrome (MERS)-CoV in 2012 [3], while the newly emergent coronavirus, initially referred to as 2019-nCoV and subsequently termed SARS-CoV-2, the disease it produces has been termed COVID-19, which causes respiratory infection and can progress to severe pneumonia and, in a small number of cases, death [4]. Although these coronaviruses were isolated from different human and animal hosts at different times and locations, they all belong to the species severe acute respiratory syndrome-related coronavirus [5, 6].

The increasing mortality rate warrants that vulnerable populations in the society be identified and protected. When COVID-19 and other CoV infect women who are pregnant, it increases the risk of adverse obstetrical and neonatal outcomes and results in severe respiratory disease [5]. Previous data from multiple studies of influenza and other respiratory infectious diseases have demonstrated an increased risk of maternal obstetrical complications when compared with nonpregnant women due to physiological changes occurring during pregnancy [7]. This association has also been previously demonstrated to occur when pregnant women became infected with either of the two pathogenic coronavirus infections (SARS-CoV 2 and MERS-CoV) [8].

Coronavirus infection in pregnant women makes clinical management more difficult by prolonging and complicating the illness and compromises the treatment [9]. Researchers are still in question regarding the transmission of the novel and previous coronavirus infection from a pregnant woman to her fetus, a process termed vertical transmission [10–12]. There are few published cases of coronavirus disease occurring during pregnancy and due to the possibility of mother–fetal vertical transmission, there is a concern that the fetuses may be at risk of congenital COVID-19 and other CoV outbreaks. Due to the alarming spread of CoV outbreaks throughout the world, a comprehensive understanding of the transmission of the virus from mother to fetus in utero like other emerging viral infections as Zika virus and Ebola virus [13, 14], that can threaten the health and survival of an infected mother and fetus is essential for effective management of the infection and treatment. Therefore, this systematic review and meta-analysis was aimed to assess the effect of coronavirus infection (SARS-CoV-2, MERS-CoV, and SARS-CoV) during pregnancy and its possibility of vertical maternal–fetal transmission.

## Methods

### Study design

A systematic review and meta-analysis was aimed to assess the effect of coronavirus infection (SARS-CoV-2, MERS-CoV, and SARS-CoV) during pregnancy and its possibility of vertical maternal–fetal transmission following the methodological framework suggested by Arksey and O’Malley [15].

### Search strategies

All relevant articles were searched without date limits using the following databases: PubMed, Web science, Embase, Google Scholar, Cochrane library, and Science Direct according to the Preferred Reporting Items for Systematic Reviews and Meta-analysis (PRISMA) [16]. All searches were limited to article written in English given that such language restriction does not alter the outcome of the systematic reviews and meta-analysis [17]. The gray literature of observational studies was searched through the review of reference lists and input of content experts. We searched scientific publications from 2003 to 2020. All papers published until the end of April 30, 2020, and fulfill the inclusion criteria were considered. The search used the following keywords: coronavirus, novel coronavirus-2019 infection, pregnancy, Middle East respiratory syndrome, severe acute respiratory syndrome, severe acute respiratory syndrome coronavirus-2, and vertical transmission. We searched all terms with the help of Boolean operators like “AND” or “OR”.

### Eligibility criteria

All articles with a report of pregnant women with confirmed severe acute respiratory syndrome coronavirus-2 (SARS-CoV-2), Middle East respiratory syndrome coronavirus (MERS-CoV) or severe acute respiratory syndrome coronavirus (SARS-CoV) and other related illness with different clinical features published in English from 2003 to April 30/2020 were included in the study. Case studies and case series reported pregnant women with confirmed coronavirus; other clinical features were included with caution. Studies pertaining to other coronavirus-related illnesses, studies that were not fully accessible, and duplicate publications of the same study were excluded.

### Assessment of study quality

Studies selected for inclusion were assessed for methodological quality by all authors’ independently using the standard critical appraisal instruments of the Joanna Briggs Institute Meta-Analysis of Statistics Assessment Review Instrument (JBI-MAStARI) [18]. The disagreements were resolved by consensus.

### Outcome measures

The primary outcome variable of this study was the pregnancy outcomes observed, listed as follows: preterm birth (PTB; either before 37 or 34 weeks of gestation), preeclampsia, preterm prelabor rupture of membranes, (pPROM), fetal growth restriction (FGR), miscarriage, maternal death, mode of delivery and other clinical feature, laboratory findings and coexisting disease. The secondary outcomes were the perinatal outcomes observed as listed: fetal distress, apgar score < 7 at 5 min, neonatal asphyxia, admission to the neonatal intensive care unit (NICU), perinatal death, including both stillbirth, and neonatal death, evidence in utero transmission.

### Data extraction and synthesis

The data were extracted using a standardized data extraction format, adapted from the Joanna Briggs Institute (JBI), by three authors (KD, EA, and AG), independently extracting all necessary data using an excel spreadsheet form. Then the extracted data were merged for systematic analysis. Any disagreements during the data extraction were resolved through discussion and consensus. The main outcomes extracted from the study were: primary author, study design, publication year, country, patient demographics, maternal and perinatal outcome, and evidence of in utero transmission of coronavirus. All clinical characteristics of pregnant women infected with coronavirus, laboratory and radiological findings, treatment options, and data on associated risk factors were extracted by the authors.

### Statistical analysis

Following data extraction, systematic review and meta-analysis were carried out using R software version 3.6.1 and STATA statistical software (version 13) with user-contributed commands for meta-analyses: metaprop, metan, metainf, metabias, and metareg [19]. The effect sizes and SEs of the studies were pooled using a random-effects model to calculate the pooled estimates of laboratory findings, other clinical features and coexisting diseases among patients with COVID-19 infection. A meta-analysis was also planned to assess the association of various comorbidities and laboratory findings with the severity of disease.

### Risk of bias and sensitivity analysis

The standard error for each original study was calculated using the binomial distribution formula. Evidence for statistical heterogeneity among reported prevalence was using the Cochrane *Q*-test and *I*^2^ statics [20]. The pooled proportion was estimated by using the back-transform of the weighted mean of the transformed proportions for both the fixed-effects model and the random-effects model [21]. A significance level of *P* < 0.10 and *I*^2^ > 50% was interpreted as evidence of heterogeneity [22, 23]. A potential source of heterogeneity was investigated by subgroup analysis and meta-regression analysis [24]. Where statistical pooling was not possible, the findings were presented in a narrative form including tables and figures to aid in data presentation where appropriate.

Sensitivity analyses were conducted to weigh up the relative influence of each individual study on the pooled effect size using a user-written function, metainf. The presence of publication bias was assessed informally by visual inspection of funnel plots [25]. Point prevalence, as well as 95% confidence intervals, was presented in the forest plot format.

## Results

### Study selection

**Fig. 1:**
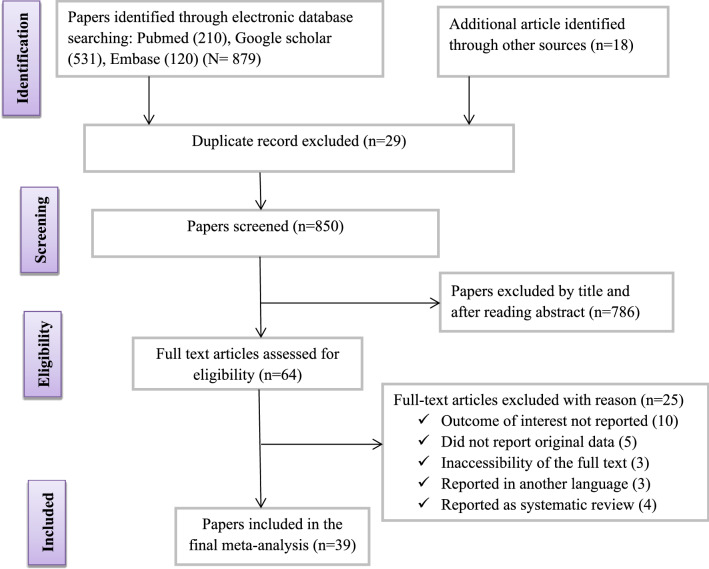
Flowchart of study selection for systematic review and meta-analysis of pregnant women with laboratory-confirmed coronavirus infection

Database searches identified a total of 879 articles. From these initial articles, 29 articles were excluded due to duplication. From the remaining 850 articles, 786 articles were excluded after review of their titles and abstracts confirmed nonrelevance to this review. Therefore, 64 full-text articles were assessed for eligibility based on the preset criteria, which resulted in the further exclusion of 25 articles primarily due to the outcome of the interest being not reported in the study. Ultimately, 39 studies met the eligibility criteria and were included in the final review (Fig. 1 and Table 1).

**Table 1:** Review of studies reporting coronavirus infection in pregnant women

Author	Year	Study area	Test design	Pregnancy (*n*)	Age group	Virus detected in mother	Severe cases *n* (%)
Chen [12]	2020	China	Retrospective	9	26–40	SARS-CoV-2	0 (0%)
Zhu [26]	2020	Wuhan	Retrospective	9	25–35	SARS-CoV-2	2 (22.2%)
Penfield [27]	2020	New York	Retrospective	11	22–40	SARS-CoV-2	5 (45.5%)
Liu [28]	2020	Wuhan	Case series	3	30–34	SARS-CoV-2	3 (100%)
Yan [29]	2020	China	Retrospective	65	24–40	SARS-CoV-2	8 (12.5%)
Zhang [30]	2020	Wuhan	Retrospective	16	24–34	SARS-CoV-2	1 (6.3%)
Pierce [31]	2020	US	Cohort study	64	n/a	SARS-CoV-2	64 (100%)
Chen [32]	2020	Wuhan	Cross-sectional	5	25–30	SARS-CoV-2	0 (0%)
Dong [33]	2020	Wuhan	Case report	1	29	SARS-CoV-2	1 (100%)
Fan [34]	2020	Wuhan	Case report	2	29,34	SARS-CoV-2	n/a
Yang [35]	2020	Wuhan	Cross-sectional	7	n/a	SARS-CoV-2	n/a
Li [36]	2020	China	Case report	1	30	SARS-CoV-2	0 (0%)
Wang [37]	2020	China	Case report	1	28	SARS-CoV-2	n/a
Cui [38]	2020	China	Case report	1	55	SARS-CoV-2	1 (100%)
Yu [39]	2020	Wuhan	Retrospective	7	29–34	SARS-CoV-2	0 (0%)
Zeng [40]	2020	Wuhan	Cohort study	33	n/a	SARS-CoV-2	n/a
Breslin [41]	2020	New York	Retrospective	43	20–39	SARS-CoV-2	6 (14.0%)
Liu [42]	2020	China	Retrospective	13	24–36	SARS-CoV-2	2 (15.4%)
Liu [43]	2020	Wuhan	Retrospective	15	23–40	SARS-CoV-2	n/a
Liu [44]	2020	Shanghai	Retrospective	16	22–42	SARS-CoV-2	n/a
Liu [45]	2020	Wuhan	Cross-sectional	10	27–34	SARS-CoV-2	n/a
Knight [46]	2020	UK	Prospective	427	n/a	SARS-CoV-2	40
Lokken [47]	2020	USA	Retrospective	46	26–34	SARS-CoV-2	n/a
Lumbreras [48]	2020	Mexico	Retrospective	308	26–39	SARS-CoV-2	n/a
Andrikopoulou [49]	2020	NewYork	Case series	158	n/a	SARS-CoV-2	34
MERS-CoV							
Assiri [50]	2016	SA	Case series	5	15–45	MERS-C0V	5 (100%)
Alfaraj [51]	2019	SA	Case series	2	29, 39	MERS-C0V	0
Jeong [52]	2017	SK	Case report	1	39	MERS-C0V	0
Park [53]	2016	SK	Case report	1	39	MERS-C0V	1 (100%)
Malik [54]	2016	UAE	Case report	1	32	MERS-C0V	1 (100%)
Payne [55]	2014	Jordan	Case report	1	39	MERS-C0V	0
Alserehi [56]	2016	SA	Case report	1	33	MERS-C0V	1 (100%)
SARS-CoV							
Robertson [57]	2004	USA	Case report	1	36	SARS-CoV	1 (100%)
Stockman [58]	2004	USA	Case report	1	38	SARS-CoV	0
Lam [59]	2004	China	Case control	10	25–37	SARS-CoV	6 (60%)
Wong [60]	2004	China	Retrospective	12	24–44	SARS-CoV	6 (50%)
Schneider [61]	2004	USA	Case report	1	–	SARS-CoV	0
Ng [62]	2006	China	Retrospective	7	25–34	SARS-CoV	3 (42.9%)
Yudin [63]	2005	Canada	Case report	1	33	SARS-CoV	0

### Description of included studies

In the current review, 39 articles with a total of 1316 pregnant women with laboratory-confirmed CoV [12, 26–49] infection [1271 with SARS-CoV-2 [12, 26–49], 12 with MERS-CoV [50–56] and 33 with SARS-CoV [57–63] (Table 1)] and reported different maternal and perinatal outcome were included in this study. Most of the studies included in this review were retrospective studies and case series as listed in Table 1. The number of pregnant women confirmed to be infected with SARS-CoV-2, MERS-CoV, and SARS-CoV in different studies ranged from 1 to 427. Majority of the studies that fulfill the inclusion criteria were from China (Table 1). The mean age of the study participants was 33.2 years with a standard deviation of 8.3 years. Out of the 39 studies, 30 papers reported severe disease among pregnant women with a prevalence of SARS-CoV-2 [20.1% (167/833)], MERS-CoV [66.7% (8/12)] and SARS-CoV [48.5% (16/33)] (Table 1).

### Commonly reported clinical feature and laboratory finding of pregnant women infected with coronaviruses (SARS-CoV-2, MERS-CoV, and SARS-CoV)

From the total of 39 included studies, only 32 studies with a total of 914 pregnant women infected with coronavirus had reported different clinical features. Meta-analysis was performed for those different clinical features of pregnant women infected with coronavirus with available data. According to the report of 39 studies, fever, cough, and fatigue were the most common clinical features of coronavirus-infected pregnant women with prevalence ranged from 30 to 67% in SARS-CoV-2, 50–78% in MERS-CoV and 80–97% in SARS-CoV. The pooled prevalence of all clinical symptoms was 26% with 95% CI (15.2–40.1). Pneumonia was the most diagnosed clinical symptom among pregnant women in the three coronavirus infections with a prevalence of 71.2% in SARS-CoV-2, 71.4% in MERS-CoV and 88.9% in SARS-CoV (Table 2). Among the most commonly reported laboratory findings, lymphocytopenia was the most frequently reported in the three coronavirus infections with prevalence ranged from 63 to 100%, followed by elevated C-reactive protein and leukopenia with prevalence ranged from 45 to 100% (Table 2).

**Table 2:** Pooled prevalence of clinical features and laboratory findings of pregnant women with coronavirus infection in the present systematic review and meta-analysis

Clinical feature of patients with CoV infection	SARS-CoV-2				MERS-CoV				SARS-CoV			
	Study (*n*)	Reports (*n*)	Pregnancies (*n*)	Pooled % (95%-CI)	Study (*n*)	Reports (*n*)	Pregnancies (*n*)	Pooled % (95%-CI)	Study (*n*)	Reports (*n*)	Pregnancies (*n*)	Pooled % (95%-CI)
Fever	19	575	870	66.1% [56.1–79.8]	6	9	12	75% [30.2–88.9]	7	32	33	96.9% [84.6–100]
Cough	18	458	869	52.7% [34.7–67.7]	4	7	9	77.8% [40.1–97.5]	6	21	26	80.8% [61.4–93.6]
Fatigue	8	192	634	30.3% [14.4–49.6]	1	1	2	50% [1.2–99.2]	–	–	–	–
Dyspnea	7	49	303	16.2% [7.6–22.3]	1	1	2	50% [1.4–99.6]	1	9	10	90% [55.3–100]
Myalgia	5	61	293	20.8% [7.5–27.8]	2	3	8	37.5 [9.3–76.2]	2	16	22	72.7% [50.1–89.8]
Shortness of Breath	6	186	586	31.7% [18.2–41.5]	1	1	2	50% [1.4–99.6]	3	9	24	37.5% [19.2–59.6]
Sore throat	6	78	668	11.7% [5.1–29.1]	–	–	–	–	2	5	22	22.7% [8.7–45.5]
Chill	3	44	480	9.2% [4.7–71.1]	–	–	–	–	3	15	23	65.2% [43.1–84.8]
Headache	5	88	674	13.1% [8.5–33.2]	1	1	2	50% [1.4–99.6]	4	15	24	62.5% [41.3–81.9]
Muscle pain	3	67	628	10.7% [4.9–25.4]	–	–	–	–	2	2	11	18.2% [3.2–35.9]
Diarrhea	9	41	753	5.4% [2.2–10.3]	1	1	2	50% [1.4–99.6]	2	3	22	13.6% [6.6–48.7]
Abdominal pain	2	5	64	7.8% [3.5–17.1]	1	1	2	50% [1.4–99.6]	–	–	–	–
Pneumonia	21	368	517	71.2% [68.6–95.8]	4	5	7	71.4% [29.5–96.2]	5	8	9	88.9% [52.1–100]
Laboratory finding												
Leukocytosis	6	27	95	28.4% [20.1–39.6]	–	–	–	–	3	10	24	41.7% [22.1–63.2]
Leukopenia	6	43	95	45.3% [35.4–56.3]	–	–	–	–	3	14	24	58.3% [37.2–78.3]
Lymphocytosis	12	8	70	11.4% [5.5–21.3]	–	–	–	–	4	3	24	12.5% [3.2–32.8]
Lymphocytopenia	13	92	146	63% [55.7–71.4]	1	1	1	100% [3.3–100]	4	20	24	83.3% [63.1–95.5]
ALT↑	3	9	48	18.8% [9.5–33.7]	2	1	3	33.3% [1.6–91.3]	2	2	8	25% [3.3–65.3]
AST↑	3	8	48	16.7% [7.2–30.5]	2	1	3	33.3% [1.6–91.3]	2	2	8	25% [3.3–65.3]
LDH↑	3	8	23	34.8% [16.2–57.9]	–	–	–	–	–	–	–	–
C-reactive protein ↑	11	80	143	55.9% [47.4–64.2]	1	1	1	100% [3.3–100]	1	1	1	100% [3.3–100]

*AST* aspartate aminotransferase, *ALT* alanine aminotransferase, *LDH* lactate dehydrogenase

### Commonly reported radiological finding and treatment of pregnant women infected with coronaviruses (SARS-CoV-2, MERS-CoV, and SARS-CoV)

The most common computed tomography imaging features in pregnant women infected with coronaviruses were ground-glass opacity followed by bilateral pneumonia with a prevalence of 65.8% and 57.9%, respectively (Table 3). In this study, ribavirin and oseltamivir were the most commonly used antiviral therapy used for the treatment of viral pathogens among pregnant women infected with coronaviruses with a prevalence of 65.2% and 56.5%, while the most common antibiotic therapy used for the treatment of common bacterial co-infection was azithromycin with prevalence of 35%. In this study, hydroxychloroquine was the leading drug used by people infected with coronaviruses with a prevalence of 79.7%. From the total coronavirus-infected pregnant women, around 78.8% were treated with oxygen therapy while 18.1% were supported by mechanical ventilation (Table 3).

**Table 3:** Pooled prevalence of radiological findings and treatment of pregnant women with coronavirus infection in the present systematic review and meta–analysis

Radiological finding and treatment of patients with CoV infection	Study (*n*)	Number of reports	Pregnancies (*n*)	Pooled % (95%-CI)
Radiological finding				
Unilateral pneumonia	2	3	15	20% [4.4–48.6]
Bilateral pneumonia	6	11	19	57.9% [33.5–80.1]
Ground-glass opacity	8	50	76	65.8% [54.2–76.4]
Multiple patchy infiltrate	5	5	22	22.7% [8.9–45.2]
Treatment				
Oseltamivir	6	14	23	56.5% [35.1–64.3]
Remdesivir	5	3	16	18.8% [13.8–29.1]
Lopinavir	2	2	12	16.7% [13.1–41.1]
Ritonavir	3	2	13	15.4% [10.1–31.7]
Ganciclovir	7	2	10	20% [12.8–44.2]
Ribavirin	11	15	23	65.2% [47.3–78.4]
Other antiviral therapy	11	89	128	69.5% [39.2–77.8]
Azithromycin	4	7	20	35% [22.3–48.5]
Other antibiotic therapy	13	102	157	64.9% [60.9–73.1]
Hydroxychloroquine	12	59	74	79.7% [70.1–89.3]
Hydrocortisone	3	11	24	45.8% [37.2–59.6]
Mechanical ventilation	7	23	127	18.1% [12.26.8]
Oxygen therapy	9	89	113	78.8% [65.4–89.6]

### The outcome of pregnant women infected with coronavirus and their newborn

Out of 1316 pregnant women infected with CoV, 46.5% give birth at > 37 weeks of gestation, while the rates of PTB < 34 and < 37 weeks of gestation were 9.5% and 14.3%, respectively. Preeclampsia was reported among 5.9% of pregnant women, while the rate of miscarriage for CoV infection was 14.5%. pPROM and FGR were rated 9.2% and 2.8%, respectively. From the total CoV-infected pregnant women, 31.3% were admitted to ICU from which 2.7% were died. The prevalence of cesarean delivery was 56.9%, while 28.6% had undergone normal delivery. Fetal distress was reported in 26.5%, while neonatal asphyxia was reported in only 1.4% of neonates. Only, 1.2% of neonates had apgar score < 7 at 5 min. Neonate admitted to ICU was rated 11.3%, while the rate of perinatal death was 2.2%. In the current review, none of the studies reported transmission of CoV from the mother to the fetus in utero during the follow-up period (Table 4).

**Table 4:** Pooled proportions of the different maternal and postnatal outcomes and coexisting disorder identified in the present systematic review

Pregnancy and perinatal postnatal outcome	Study (*n*)	Number of reports	Pregnancies or neonate (*n*)	Pooled % (95%–CI)
Pregnancy outcome				
PTB < 34 week	25	68	719	9.5% [7.1–39.5]
PTB < 37 week	22	91	637	14.3% [10.2–30.2]
> 37 week	25	330	709	46.5% [41.2–56.3]
Pre-eclampsia	9	10	169	5.9% [3.6–11.7]
pPROM	11	17	183	9.3% [6.6–14.8]
FGR	14	3	108	2.8% [1.8–8.9]
Miscarriage	4	9	62	14.5% [7.7–26.5]
ICU admission	29	66	211	31.3% [27.2–55.5]
Maternal death	11	15	557	2.7% [1.4–14.6]
Cesarean delivery	32	440	772	56.9% [48.2–78.9]
Normal delivery	17	198	692	28.6% [22.4–41.6]
Prenatal outcome				
Fetal distress	17	18	68	26.5% [17.5–39.5]
Apgar score < 7	16	1	81	1.2% [0.0–7.7]
Neonatal asphyxia	11	1	71	1.4% [0.0–8.9]
Admission to NICU	8	9	73	11.3% [5.6–20.3]
Perinatal death	21	10	452	2.2% [1.2–12.8]
Vertical transmission	39	0	1316	0.0% [0.0–1.0]
Coexisting disorders				
Hypertension	7	81	954	8.5% [7.1–17.4]
Gestational diabetes	9	85	881	9.6% [6.6–20.7]
Cardiovascular disease	2	27	470	5.7% [3.0–12.1]
Digestive disease	3	4	111	3.6% [1.1–9.8]
Asthma	4	44	794	5.5% [2.3–36.4]

*PTB* preterm birth, *pPROM* preterm premature rupture of membranes, *FGR* fetal growth restriction, *ICU* intensive care unit, *NICU* neonatal intensive care unit

In our systematic review, different comorbidities that aggravate the infection were found among pregnant women infected with CoV. From the total CoV-infected pregnant women, the rate of gestational diabetes was 9.6%, while hypertension was reported in 8.5% of pregnant women infected with CoV. The rate of asthma in pregnant women infected with CoV was 5.5%, while cardiovascular disease and digestive disease rated 5.7% and 3.6%, respectively (Table 4).

### Comparison of severe cases among coronavirus infection (SARS-CoV-2, MERS-CoV, and SARS-CoV)

**Fig. 2:**
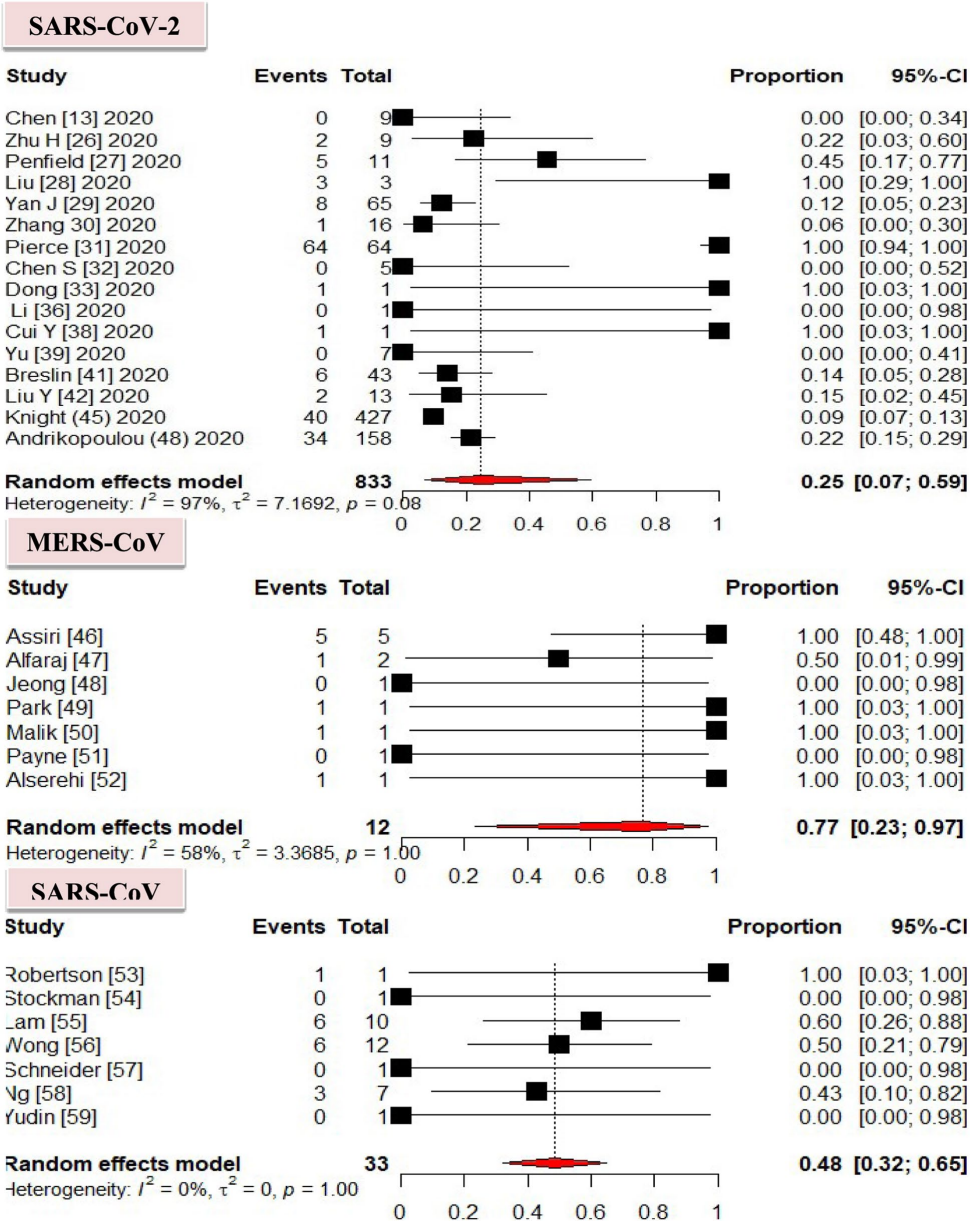
Pooled proportions of severe cases in the overall population of pregnancies infected with coronavirus infection

In this meta-analysis, MERS-CoV was the most predominant causative agent of severe cases among infected pregnant women with a prevalence of 77% with 95% CI [23–97], followed by SARS-CoV rated 48% with 95% CI [32–65]. According to our findings, SARS-CoV-2 was the least causative agent of severe cases among the infected pregnant women, which rated 25% with 95% CI [7–59] (Fig. 2).

### The effect of SARS-CoV-2, MERS-CoV, and SARS-CoV in pregnant women and their newborns

#### SARS-CoV-2

Out of 39 eligible articles, 25 studies [12, 26–49] reported information on infections caused by SARS-CoV-2 among a total of 1271 pregnancies. The prevalence of SARS-CoV-2 among preterm birth at < 37 and 34 weeks of gestation was 14.3% and 8.9%, respectively, while 46.2% of pregnant women give birth at > 37 weeks of gestation. Preeclampsia was reported among 5.7% of pregnant women with COVID-19. pPROM was reported in 8.9%, while the rate of fetal growth restriction was reported in 1.2%. In this study, miscarriage was rated 2.4%. ICU-admitted pregnant women were accounted for 28.5%, while the rate of maternal death was reported in 1.5%. The prevalence of cesarean delivery was 57% (Table 5). Fetal distress was reported among 25%, while the rate of neonatal asphyxia was 1.6%. The prevalence of Apgar score < 7 at 5 min was 1.4%. The rate of newborns admitted to NICU was 11.6% in which perinatal death was reported among 2.9%. None of the studies reported transmission of SARS-CoV-2 from the mother to the fetus in utero during the follow-up period (Table 5).

**Table 5:** Pooled proportions of the different pregnancy and perinatal outcomes identified in the present systematic review

Pregnancy outcome	SARS-CoV-2				MERS-CoV				SARS-CoV			
	Study (*n*)	Reports (*n*)	Pregnancies or neonate (*n*)	Pooled % (95%-CI)	Study (*n*)	Reports (*n*)	Pregnancies or neonate (*n*)	Pooled % (95%-CI)	Study (*n*)	Reports (*n*)	Pregnancies or neonate (*n*)	Pooled % (95%-CI)
PTB < 34 week	11	61	682	8.9% [6.1–19.3]	7	4	12	33.3% [14.2–38.9]	7	3	25	12% [3.6–31.5]
PTB < 37 week	9	86	602	14.3% [9.2–33.2]	7	0	12	0.0% [0.0–7.4]	6	5	23	21.7% [7.4–44.6]
>37 week	13	317	686	46.2% [41.5–58.9]	5	8	10	80% [44.2–97.8]	7	5	13	38.5% [0.0–68.4]
Preeclampsia	5	9	159	5.7% [3.3–10.1]	2	1	8	12.5% [0.0–53.6]	2	0	2	0% [0.0–84.7]
pPROM	7	16	179	8.9% [5.5–14.6]	2	0	2	0.0 [0.0–84.2]	2	1	8	12.5% [0.0–53.8]
FGR	4	1	86	1.2% [0.0–6.3]	4	0	6	0.0% [0.0–46.5]	6	2	16	12.5% [2.2–38.6]
Miscarriage	2	1	41	2.4% [0.0–13.5]	–	–	–	–	2	8	21	38.1% [18.7–62.5]
ICU admission	25	53	186	28.5%23.1–54.4]	2	1	3	33.3% [1.2–91.3]	2	12	22	54.5% [32.1–76.8]
Maternal death	4	8	523	1.5% [1.2–9.6]	4	4	10	40% [12.5–74.3]	3	3	24	12.5% [3.3–32.9]
Cesarean delivery	19	426	747	57.0% [48.9–78.8]	6	6	9	66.7% [30.2–93.5]	6	8	16	50% [25.6–75.9]
Normal delivery	11	192	662	29.0% [21.4–33.5]	2	2	12	16.7% [2.4–48.6]	4	4	18	22.2% [6.6–48.7]
Perinatal outcome												
Fetal distress	7	14	56	25% [14.4–39.6]	5	0	0	0.0% [0.0–52.3]	5	4	12	33.3% [10.1–65.2]
Apgar score < 7	7	1	72	1.4% [0.0–7.8]	4	0	4	0.0% [0.0–60.1]	5	0	5	0.0% [0.0–52.3]
Neonatal asphyxia	4	1	64	1.6% [0.0–8.9]	3	0	3	0.0% [0.0–71.4]	4	0	4	0.0% [0.060.8]
Admission to NICU	4	8	69	11.6% [5.4–22.6]	3	1	3	33.3% [1.3–84.5]	1	0	1	0.0% [0.0–98.5]
Perinatal death	8	5	430	1.2% [1.0–8.7]	7	4	12	33.3% [10.6–65.3]	6	1	10	10% [0.0–45.3]
Vertical transmission	25	0	1271	0.0 [0.0–1.5]	7	0	12	0.0% [0.0–26.5]	7	0	7	0.0% [0.0–41.6]

*PTB* preterm birth, *pPROM* preterm premature rupture of membranes, *FGR* fetal growth restriction, *ICU* intensive care unit, *NICU* neonatal intensive care unit

#### MERS-CoV

Out of 35 eligible articles, seven studies [50–56] reported information on 12 pregnant women infected with MERS-CoV. The prevalence of preterm birth among pregnant women of < 34 weeks of gestation was 33.3%, while 80% of pregnant women give birth at > 37 weeks of gestation. Preeclampsia was observed among 5.7% of pregnant women infected with MERS-CoV. ICU-admitted pregnant women accounted for 33.3%, while the rate of maternal death was reported to be 40%. The prevalence of pregnant women given birth by cesarean was 66.7%, while 16.7% of pregnant women underwent normal delivery. Perinatal death was reported among 33.3% of the newborns, while none of the studies reported fetal distress, apgar score < 7 at 5 min, neonatal asphyxia, and admission to the neonatal intensive care unit. None of the studies reported transmission of MERS-CoV from the mother to the fetus in utero during the follow-up period (Table 5).

#### SARS-CoV

Out of 35 eligible articles, seven studies [57–63] reported information on 33 pregnant women infected with SARS-CoV. The prevalence of SARS-CoV among preterm birth < 37 and 34 weeks of gestation was 21.7% and 12%, respectively, while 38.5% of pregnant women give birth at > 37 weeks of gestation. pPROM was reported in 12.5%, while the rate of fetal growth restriction was reported in 12.5%. Miscarriage was reported among 38.1% of pregnant women. ICU admitted pregnant women were accounted for 54.5%, while the rate of maternal death was reported in 12.5%. The prevalence of pregnant women giving birth by cesarean was 50%, while 22.2% of pregnant women underwent normal delivery. The prevalence of fetal distress and perinatal death was 33.3% and 10%, respectively, while none of the studies reported apgar score < 7 at 5 min, neonatal asphyxia and admission to the neonatal intensive care unit. None of the studies reported transmission of SARS-CoV from the mother to the fetus in utero during the follow-up period (Table 5).

### Heterogeneity and risk of bias

Subgroup analysis was conducted to justify the cause of heterogeneity. Subgroup analysis of the included studies showed that the possible cause of heterogeneity was a sample size difference, especially in SARS-CoV-2 (*I*^2^ = 94%). The funnel plots suggest a publication bias for some of the study of the parameters (*P* < 0.02).

## Discussion

### Summary of findings

We conducted a systematic review and meta-analysis to provide an overview of the effect of CoV (SARS-CoV-2, MERS-CoV, and SARS-CoV) infection on maternal and perinatal, and the possibility of vertical transmission of the virus from pregnant women to the fetus. The rapidly spreading nature of COVID-19 and previous CoV infections could have a significant effect on human health. Thus, attention should be given to pregnant women, and they should be included in preparedness and response plans. In previous outbreaks, clinicians did not volunteer to treat or vaccinate pregnant women because of concerns for fetal safety [64]. The pregnant state, with alterations in hormone levels and decreased lung volumes due to a gravid uterus and slightly immunocompromised state may predispose patients to a more rapidly deteriorating clinical course and face a greater risk of harm if they get respiratory infections.

In the current study, the predominant signs and symptoms of hospitalized pregnant women suffering from COVID-19 and other coronavirus infections were viral pneumonia, fever, cough, fatigue, and myalgia. The Centre for disease control listed these symptoms to be the leading clinical feature of patients infected with COVID-19 and other coronaviruses [65]. The high fever after delivery may have resulted from immunity reduction due to fatigue and blood loss in childbirth, anatomy of female genitalia, sweating during puerperium, and postpartum lactation. Once postpartum fever occurred, obstetricians and gynecologists should follow and perform a differential diagnosis to exclude breast swelling, mastitis, urinary tract infections, common colds, and reproductive tract infections must be focused on. Gastrointestinal symptoms like diarrhea and abdominal pain were also observed in pregnant women with COVID-19 and other coronavirus infections. Gestational diabetes and hypertension were the most common coexisting disorders with a prevalence of 9.6% and 8.5%. More than half of the pregnant women infected with coronaviruses were treated by antiviral and antibiotic therapy.

In our study, the most frequently reported laboratory findings was lymphocytopenia (66.1%), followed by elevated C-reactive protein marker (56.6%), leukopenia (48.3%) and elevated lactate dehydrogenase (34.8%). This is similar to previous studies reporting on COVID-19 and other CoV infections [66–69]. Laboratory findings like leukopenia and lymphocytopenia were helpful to distinguish viral infections from common bacterial infections [70]. The most common computed tomography imaging features in pregnant women with COVID-19 and non-COVID-19 pneumonia were ground-glass opacity and bilateral pneumonia with a prevalence of 57.9% and 65.8%, respectively. The pathological basis of these changes could be due to inflammatory cell infiltration and interstitial thickening, cell exudation, and hyaline membrane formation. The most common treatment options used were hydroxychloroquine (79.7%), ribavirin (65.2%), oseltamivir (56.5%) and azithromycin (35%) to treat common bacterial pathogens when secondary infections occurred and treat viral pathogens. Among CoV-infected pregnant women, oxygen therapy was 78.8%.

Based on the findings of the present study, higher prevalence of COVID-19 and other CoV was reported among preterm birth < 37 weeks of gestation. According to some studies report, infection with COVID-19 during pregnancy can cause complications for both the mother and the fetus [59, 71]. This includes preeclampsia, preterm premature rupture of membranes, fetal growth restriction, and miscarriage. Large numbers of CoV-infected pregnant women with severe cases were admitted to the intensive care unit which even resulted in the death of some mothers. Cesarean deliveries occurred among three-fourths of pregnant women infected with CoV. Regarding the perinatal outcome, fetal distress and neonatal asphyxia were the most commonly reported abnormalities in newborn. The rate of newborns admitted to the neonatal intensive care unit was around 11.3% while 2.2% of them died. MERS-CoV was the predominant causative agent of severe cases in infected pregnant women.

There is much controversy relating to the possibility of in utero transmission of SARS-CoV-2, MERS-CoV, and SARS-CoV. Multiple samples were obtained at the time of labor and delivery to test for the presence of coronavirus by qRT-PCR, including amniotic fluid aspirated from the vagina during labor, cord blood, and segments of the umbilical cord, fetal membranes, and placenta, neonatal nasopharyngeal and throat swabs, gastric aspirate and meconium samples tested negative for the coronaviruses, suggesting there was no evidence of vertical transmission in women who developed COVID-19 and non-COVID-19 coronavirus pneumonia in late pregnancy [12, 63, 72]. Studies conducted in London [73] reported a neonate born to a pregnant woman with COVID-19 that tested positive for SARS-CoV-2 in the pharyngeal swab sample 36 h after birth, it was subsequently confirmed that qRT-PCR testing of the placenta and cord blood was negative for SARS-CoV-2, it is believed that the mother or a family member transmitted the infection to the infant through close contact after delivery, not in utero through placenta [39, 74].

In the current study, the systematic testing procedures for coronavirus infection, including chest radiograph and serial RT-PCR assays with multiple clinical samples did not demonstrate the presence of SARS-CoV-2, MERS-CoV and SARS-CoV in the newborn. CoV antibody tests were performed with mother and newborn sera. In some mothers’, sera immunoglobulin G was detected by using serological tests. However, CoV antibodies for IgG, IgM, and IgA were detected in none of the newborn’s blood samples [55, 75]. Therefore, there is no evidence of intrauterine transmission of SARS-CoV-2 and other CoV from mother to newborn infants. This may be due to a very low expression of angiotensin-converting enzyme 2 (ACE2) in early maternal to fetal interface cells [76]. The virus can be transmitted through close contact or droplets to a new born after birth [73]. Thus, mothers and their neonates should be taken care of in isolated rooms to prevent neonatal transmission and effective protection measures should be implemented during delivery and postdelivery care to prevent transmission of the virus from mothers to the newborn.

### Limitations of the study

Only English articles or reports were considered for this review. The small number of cases in some of the included studies, the study design, and the lack of standardized criteria were the major limitations of this systematic review. Additionally, there is a possibility that some patients were included in more than 1 report, although all authors independently reviewed all the included studies, carefully focusing on the different institutions reporting outcomes. We included case reports and case series, thus facing a higher risk of publication bias, which could affect the estimated outcome. Furthermore, lack of denominator in case series used in this review is the other major factor that affects the estimated outcome. Moreover, when focusing on the outcomes of COVID-19 infection, and particularly perinatal outcomes, reported data are intuitively limited to a very short-term follow-up period and thus infections that occurred proximate to the delivery. This has the potential to overestimate the magnitude of risks such as PTB and underestimate more longitudinal risks such as FGR. In some of the studies, we did not find standardized criteria and timing of delivery of pregnancies affected by CoV infection.

## Conclusion

In general, based on the published data collected, fever, cough, and myalgia were the most common clinical features, while the predominant abnormal laboratory findings reported were lymphocytopenia and C-reactive protein. Bilateral pneumonia and ground-glass opacity were the most common radiological abnormal findings. Oxygen therapy was the most common treatment option used while bacterial coinfection was treated by antibiotics therapy, and viral pathogen was treated by antiviral therapy. Among the coronavirus species, MERS-CoV was the leading cause of severe cases in infected pregnant women. Pregnant women infected with coronaviruses are at increased risk of adverse obstetrical outcomes, compared with the general population. The infection outcome was mainly associated with a relatively higher rate of cesarean delivery, preterm birth, intensive care unit admission, preeclampsia, miscarriage, fetal distress, and perinatal death. None of the studies reported transmission of CoV from the mother to the fetus in utero. This may be due to a very low expression of angiotensin-converting enzyme 2 (ACE2) in early maternal–fetal interface cells as suggested by different experts.

## Data Availability

All data relevant to the study are included in the article.

## Abbreviations

CoV: Coronavirus
FGR: Fetal growth restriction
ICU: Intensive care unit
JBI: Joanna Briggs Institute
MERS-CoV: Middle East respiratory syndrome coronavirus
NICU: Neonatal intensive care unit
pPROM: Preterm prelabor rupture of membranes
PTB: Preterm birth
RT-PCR: Real-time polymerase chain reaction
qRT-PCR: Quantitative real-time polymerase chain reaction
SARS-CoV: Severe acute respiratory syndrome coronavirus
